# *Theileria annae* in a young Swedish dog

**DOI:** 10.1186/1751-0147-55-50

**Published:** 2013-07-10

**Authors:** Ulrika Falkenö, Séverine Tasker, Eva Osterman-Lind, Harold W Tvedten

**Affiliations:** 1 Clinical Pathology Laboratory, University Animal Hospital, Swedish University of Agricultural Sciences, Uppsala, Sweden; 2 Acarus Laboratory, Molecular Diagnostic Unit, Langford Veterinary Services, School of Veterinary Sciences, University of Bristol, Langford, Bristol, UK; 3 National Veterinary Institute of Sweden, Department of Virology, Immunobiology and Parasitology, Section for Parasitological Diagnostics, Uppsala, Sweden; 4 Department of Clinical Sciences, Faculty of Veterinary Medicine and Animal Sciences, Swedish University of Agricultural Sciences, Uppsala, Sweden

**Keywords:** Babesiosis, Dog, Anemia

## Abstract

A severe regenerative anemia was detected in a 12-week-old mixed breed puppy in Sweden.

A small protozoan parasite was observed in erythrocytes on a blood smear. It was initially suspected to be *Babesia gibsoni* based on its size and because *B. gibsoni* was previously recorded in Sweden.

Surprisingly, specific polymerase chain reaction analysis identified the protozoan as *Theileria annae*. *T. annae* is endemic in Northwest Spain, is very uncommonly reported elsewhere and has never been recorded in Scandinavia. *T. annae* has been identified in dogs used for dog fighting, and it is thought to be transmitted by dog bites. This puppy was a mixed pit bull terrier. Pit bull terriers are sometimes used for dog fighting. *T. annae* has been reported to be transmitted vertically, and in light of the puppy’s age, this transmission was suspected in the present case.

## Background

Several blood parasites, such as *Babesia spp.*, infect erythrocytes in dogs and may lead to hemolytic anemia. Babesiosis in domestic dogs and wild canids is caused by several vector-borne, geographically widespread hemoprotozoan parasites belonging to the *Babesia* and *Theileria* genera, and the most commonly reported species in Europe include *B. canis*, *B. gibsoni*, *B. vogeli* and *T. annae*[1,2]. *T. annae* is also called *Babesia* microti-like or Spanish isolate/agent because it is endemic in Galicia, Northwestern Spain. *T. annae* has also been identified in Portugal, Croatia and in one dog in North America [2-7]. *Ixodes hexagonus* is the likely vector of *T. annae* in Northwest Spain, but other ticks such as *Rhipicephalus sanguineus*, *Ixodes ricinus* and *Dermacentor* spp. have also been proposed as possible transmitters [2,8,9]. A vertical (transplacental) transmission of *T. annae* is also likely as it has been detected in a German shepherd bitch and her 2-month old pup [7]. *T. annae* has also been detected in a dog confiscated from dogfighting operations in North America suggesting that *T. annae* may be transmitted by dog bites as occurs with *B. gibsoni*[6].

*T. annae* causes severe illness, and dogs may present with weakness, fever, lethargy, hemoglobinuria, tachycardia and tachypnea [2]. Laboratory findings in dogs from Northwest Spain include moderate to severe regenerative anemia (hematocrit below 31%), with marked reticulocytosis. Moderate to severe thrombocytopenia is a common finding, with 50% of the dogs having platelet counts below 23 × 10^9^/L. Leukocytosis and evidence of hepatic disease are uncommon. Azotemia has been recorded in 10–36% of dogs infected with *T. annae* at the time of diagnosis, which is a poor prognostic marker, with a 22% fatality rate within a week post diagnosis [5,10,11].

*T. annae* appears as a small (less than 2 μm), single, ring-shaped intraerythrocytic piroplasm in blood smears from infected canids. Mostly, the parasitemia is of low level [5,12]. Specific diagnosis of the parasite is confirmed by polymerase chain reaction (PCR) analysis of blood samples. 18S RNA gene has been used for conventional and nested PCR [5,6,11,12]. A reverse line blot (RBL) assay has recently been developed that simultaneously detects and separates the major vector-borne dog pathogens, including *T. annae*, in Southern Europe and the Middle East [4].

*T. annae* appears to be resistant and poorly responds to imidocarb dipropionate (Imizol, at 5–6.6 mg/kg) [2,11]. Therefore, other drugs, such as Epoximicin and Artesunate, are being investigated as potential therapeutics [13,14]. *T. annae* infection in dogs has been hypothesized to be lifelong [1]. Unless *I. ricinus* could serve as a vector, *T. annae* is not likely to become endemic in Sweden because of an absence of a suitable vector. However, global warming and increased dog traveling and importation may potentially change the distribution patterns of European tick populations, potentially leading to spread of infectious agents, such as *T. annae*, to Scandinavia.

The purpose of this first report of *T. annae* infection in a dog in Scandinavia is to alert Scandinavian veterinarians of the presence of this infection. It should be a differential diagnosis in dogs with regenerative, hemolytic anemia. This first case report of *T. annae* infection was a 12-week old Swedish, part pit bull puppy with severe regenerative anemia. It was unexpected to find this hemoprotozoan in such a young, Swedish dog and the parasite species had not been diagnosed in Scandinavia previously.

## Case presentation

A 12-week-old, mixed pit bull, male dog was presented to the University Animal Hospital of the Swedish University of Agricultural Sciences, January 2^nd^ 2013, with a history of suddenly appearing lethargic. The puppy was acquired in Sweden four weeks previously and reportedly was not in any contact with dogs which had traveled out of Sweden. It had appeared normal and active until the day before presentation. There was no history of previous illness or medication. The dog had been vaccinated at 8 weeks of age, presumably against canine distemper, parvovirus and infectious canine hepatitis. Physical examination indicated pale mucous membranes, a mild holosystolic heart murmur, mild peripheral lymphadenomegaly and suspected splenomegaly. The owner did not reported any problem with the dog’s mother.

A blood sample for complete blood count (CBC) was collected in EDTA, and analyzed within two hours (Advia 2120 hematology system, Siemens Healthcare Diagnostics, Erlangen, Germany) and blood smears were prepared, air-dried, stained with Giemsa and evaluated microscopically (Table 1). Consequently, the dog was diagnosed with a severe, moderately regenerative, macrocytic, hypochromic anemia.

**Table 1:** **Hematology data from a puppy with proven *Theileria annae *infection**

**Parameter**	**Result**	**Unit**	**Reference interval**
Erythrocytes	1.2	× 10^12^/L	(5.4-8.5)
Hemoglobin concentration	31	g/L	(132–19)
Hematocrit	0.10	L/L	(0.38-0.57)
Mean corpuscular volume	87	fl	(64–74)
Mean corpuscular hemoglobin concentration	298	g/L	(335–363)
Red cell distribution width	25.2	%	10.6-14.3
Reticulocytes	271	× 10^9^/L	(11–111)
Leukocytes	20.8	× 10^9^/L	(5.8-16)
Segmented neutrophils	12.9	× 10^9^/L	(3.3-10.4)
Band neutrophils	1.7	× 10^9^/L	(0.0-0.5)
Lymphocytes	4.0	× 10^9^/L	(1.5-4.7)
Eosinophils	0.4	× 10^9^/L	(0.1-1.2)
Monocytes	1.9	× 10^9^/L	(0.1-1.0)

Note the reference values are for adult dogs and not for 12 week old, mixed breed dogs.

**Figure 1:**
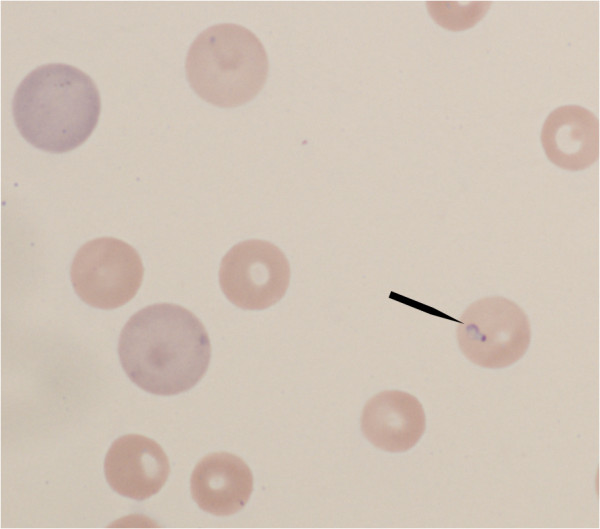
**Blood smear photomicrograph; Blood smear photomicrograph with Giemsa stain illustrates anisocytosis, 2 polychromatophils and 1 erythrocyte with *Theileria annae *(arrow).** Original magnification 1000 ×.

**Figure 2:**
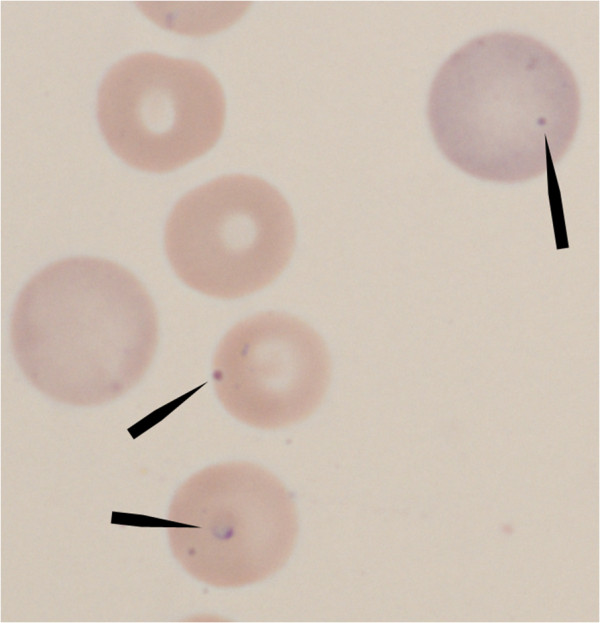
**Giemsa stained blood smear photomicrograph; Giemsa stained blood smear photomicrograph shows a *Theileria annae *(arrows) in the two lower center erythrocytes and one in the upper right polychromatophilic erythrocyte.** The lowest cell has a protozoon with more obvious cytoplasm. The protozoa have a similar sized, dark, round nucleus. Original magnification 1000 ×.

A small protozoan parasite was identified in approximately 2% of the erythrocytes. It was approximately 1–2 μm in diameter and appeared as a dark dot (nucleus) with a variable amount of visible cytoplasm (Figures 1 and 2). Based on its small size, and because *B. gibsoni* was recorded previously in Sweden, the protozoan was initially assumed to be *B. gibsoni*. No spherocytes, autoagglutination or signs of oxidative damage such as Heinz bodies or eccentrocytes were observed on blood smear evaluation. There was no apparent hemolysis in EDTA-plasma. The puppy had a mild neutrophilia with a left shift indicating inflammation. The neutrophils did not have any toxic changes. Platelet aggregates were observed on blood smear examination, precluding an accurate automated platelet count, which recorded thrombocytopenia. Visual estimation from the blood smear suggested a mild thrombocytopenia.

A sample for serum chemistry was also collected and analyzed (Architect c4000, Abbott Laboratories, Abbott Park, Illinois, USA). Serum chemistry analyses of hepatic and renal parameters as well as albumin and protein concentrations were unremarkable.

An EDTA blood sample was submitted to the Acarus Laboratory, Molecular Diagnostic Unit, Langford Veterinary Services, Bristol, UK, for molecular identification of the protozoan parasite. DNA was extracted from 100 μl of EDTA blood using the Nucleospin 8 Blood kit (Machery-Nagel, Düren, Germany), according to the manufacturer’s instructions, with elution into 100 μl elution buffer. PCR was then performed using 1 X Qiagen HotStarTaq Master Mix (Qiagen, Crawley, UK), 200nM each of primers (Metabion International AG, Martinsried, Germany) designed to amplify a 450 base pair (bp) product of the 18S rRNA gene of piroplasmas (PiroA1 Forward 5′agg-gag-cct-gag-aga-cgg-cta-cc3′and PiroB Reverse 5′tta-aat-acg-aat-gcc-ccc-aac3′), 5 μl of DNA, made up to final volume of 25 μl with RNase free water. PCR was carried out in a PTC-225 thermal cycler (MJ Research, St. Bruno, Canada) with the following protocol: 95°C 15 min, 40 × 95°C 15 sec, 60°C 20 sec and 72°C 30 sec. PCR products were analysed on a 1.5% Tris acetate-EDTA agarose (Appleton scientific Molecular Grade Agarose) gel with molecular weight markers (BioLine Easy Ladder I, Bioline Reagents Ltd, London, UK). Negative (sterile water) and positive (canine blood confirmed to be infected with *Babesia canis*) controls were also subjected to DNA extraction and PCR, and were appropriately negative and positive. The PCR product produced using the DNA extracted from the puppy was then purified using the Machery-Nagel Nucleospin Extract II kit according to the manufacturer’s instructions and the DNA quantified for sequencing using the Invitrogen Quant-iT dsDNA Assay kit & the Invitrogen Qubit Flurometer according to manufacturer’s instructions. Ten ng of purified PCR product was sent for DNA sequencing to DNA Sequencing & Services (Dundee, UK), using the PiroA1 Forward and PiroB Reverse primers. The sequence data derived was then BLAST searched (337 bp with the forward primer, 316 bp with the reverse primer) and showed 100% identity with canine *T. annae* (JX454779.1 Genbank accession no.). There was no evidence of other concurrent piroplasma infections.

The puppy was euthanized soon after initial diagnosis at the owner’s request due to the potential for lifelong infection. The owner declined a postmortem examination. Attempts to contact the dog breeder failed, but Swedish health officials are still making efforts to trace the origin of this infection.

## Conclusions

To the authors’ knowledge this is the first case of *T. annae* infection in Sweden.

This report should alert Scandinavian veterinarians that *T. annae* infection in dogs should be included as differential diagnosis in cases of regenerative anemia in this region. We can hypothesize, based on the puppy’s young age that the disease was transmitted vertically from its mother. Additionally because *T. annae* may be transmitted by bites from an infected dog and because the patient was part pit bull terrier, a breed used for dog fighting, we can also hypothesize the mother may have been infected by a bite wound. However, information on this possibility is not available.

## Competing interests

The authors declare that they have no competing interests.

## Authors’ contributions

UF has assembled most of the material for this article and written the majority of the text. ST oversaw and described the diagnostic PCR analysis and sequencing, and critically revised the article. EOL coordinated specific identification of the parasite, obtained references and critically revised the article. HT made the first diagnosis from the blood smear, photographed the blood smear and critically revised the article. All authors read and approved the final manuscript.
